# Neural Signature of Buying Decisions in Real-World Online Shopping Scenarios – An Exploratory Electroencephalography Study Series

**DOI:** 10.3389/fnhum.2021.797064

**Published:** 2022-02-14

**Authors:** Ninja K. Horr, Keren Han, Bijan Mousavi, Ruihong Tang

**Affiliations:** Brain Intelligence Neuro-Technology Ltd., Beijing, China

**Keywords:** decision-making, purchase choice, online shopping, ecological validity, EEG, time-frequency analysis, frontal alpha asymmetry, frontal theta

## Abstract

The neural underpinnings of decision-making are critical to understanding and predicting human behavior. However, findings from decision neuroscience are limited in their practical applicability due to the gap between experimental decision-making paradigms and real-world choices. The present manuscript investigates the neural markers of buying decisions in a fully natural purchase setting: participants are asked to use their favorite online shopping applications to buy common goods they are currently in need of. Their electroencephalography (EEG) is recorded while they view the product page for each item. EEG responses to pages for products that are eventually bought are compared to those that are discarded. Study 1 repeats this procedure in three batches with different participants, product types, and time periods. In an explorative analysis, two neural markers for buying compared to no-buying decisions are discovered over all three batches: frontal alpha asymmetry peak and frontal theta power peak. Occipital alpha power at alpha asymmetry peaks differs in only one of the three batches. No further significant markers are found. Study 2 compares the natural product search to a design in which subjects are told which product pages to view. In both settings, the frontal alpha asymmetry peak is increased for buying decisions. Frontal theta peak increase is replicated only when subjects search through product pages by themselves. The present study series represents an attempt to find neural markers of real-world decisions in a fully natural environment and explore how those markers can change due to small adjustments for the sake of experimental control. Limitations and practical applicability of the real-world approach to studying decision-making are discussed.

## Introduction

The question of how humans weigh up alternatives and choose between options is of widespread interest, both for multiple academic disciplines and in the praxis. To understand, predict, and influence human behavior, we need to gain deeper insights into the principles and processes underlying decision-making. While thinkers of all times have wondered about the nature of human decisions, it is a much more recent development that scientists approach the decision-making process empirically, focusing not on how we theoretically should but how we actually do make decisions [refer to Bradley (2018) on philosophical and Takemura (2004) on behavioral decision theory].

To ensure sufficient experimental control, decision scientists most commonly investigate the characteristics of simple decisions, that is, choices with a limited number of alternatives and a limited number of outcomes that follow the decision in a deterministic fashion or with predefined probabilities (refer to Houser and McCabe, 2014). Typical experimental decision-making tasks are, for example, two alternative forced choice tasks, which can be used to manipulate and model evidence accumulation toward the final decision (e.g., Smith, 1982; Ratcliff et al., 2018; Van Maanen et al., 2021), gambling tasks, which allow researchers to study how humans optimize their choice behavior through trial-and-error learning of reward structures (e.g., Toplak et al., 2010; Chiu et al., 2018), or social decision-making tasks, which investigate how humans interact and compete in decision-making (e.g., Weber et al., 2004; Rilling and Sanfey, 2011). There is a substantial amount of work devoted to methods and process models for describing the many types of choices that come from such standardized paradigms (refer to Johnson and Ratcliff, 2014 for an overview).

While empirical models of simple decision-making have certainly increased our understanding of the nature of human choice behavior, they suffer from one fundamental flaw: They are based on experiments constructed to manipulate and explain particular aspects of the decision process in an otherwise controlled situation. However, human decision strategies are highly volatile in response to small changes of contextual factors (e.g., Keren and Willemsen, 2008; Koop and Johnson, 2011; Evans and Brown, 2017; van Hoorn et al., 2019), which can result in increasingly specific models being fit to increasingly specific standardized decision situations. Relating these models to practical everyday life decision-making devoid of any experimental protocol, however, can be challenging (e.g., refer to Houser and McCabe, 2014).

To understand the biological underpinnings of decision-making, cognitive neuroscientists adapt established experimental paradigms from the behavioral sciences to investigate which brain mechanisms are related to decision behavior. This research has given interesting insights into brain areas and processes underlying important aspects of making a choice. For example, the ratio between left and right frontal activation represents an approach or an avoidance signal that can guide our actions (Smith et al., 2017; Reznik and Allen, 2018), dopaminergic neurons in the midbrain, and their connection to the orbitofrontal cortex exhibit signaling properties that can be directly related to the subjective value of decision alternatives (Padoa-Schioppa and Cai, 2011; Schultz, 2013; Rich and Wallis, 2016), and neural response patterns in the dorsolateral prefrontal cortex are crucial for the weighing up and integration of decision alternatives (Krawczyk, 2002; Lin et al., 2020; Vaidya and Fellows, 2020). Such findings combined with knowledge about neural signaling on the molecular level further inform and put practical constraints on behavioral models of decision-making (Johnson and Ratcliff, 2014). Recent decision science further shows strong advances in the development of computational models, which go far beyond original simplistic algorithms and are more and more able to account for complex decision scenarios and adaptive learning of appropriate choice behavior (Collins and Shenhav, 2022).

Research on the neural building blocks of decision-making is essential to help us understand the decision process from a biological, behavioral, and economic perspective. However, to date, its practical applicability has been limited by traditional experimental setups being too far removed from real-life decision-making. For complex real-life decisions, there are too many alternatives; time is too short and human cognitive capacity is too limited to assign all relevant subjective values, weigh up all options, and follow a particular conscious strategy. This may explain why economics, a field with an obvious interest in understanding real-life choices, initially showed little interest in the psychological and neurobiological attempts to understand human decision-making and stuck with a normative (“How should we make decisions?”) rather than a descriptive (“How do we make decisions?”) approach (Caplin and Glimcher, 2014). However, when applied to real-world judgments, economists discovered that models based on the optimal option often fail to hold true, since people behave surprisingly irrational in many situations (Tversky and Kahneman, 1974; Kahneman and Tversky, 2013; Tomer, 2015). Especially in the field of economics, decisions are often based on cognitive biases rather than rationality, for example, determining perceived value and thereby buying choices *via* inherently unrelated aspects like price (Victor and Dominic, 2021). With psychologists’ and neuroscientists’ increasing motivation to understand complex real-world decision problems and economists’ recognition that human behavior cannot fully be explained based on rationality, the two fields grew closer together, resulting in the birth of a new discipline termed neuroeconomics (Caplin and Glimcher, 2014).

Since the emergence of neuroeconomics, a lot of remarkable work has been done to shed light on decision-making with more realistic scenarios in mind. Here, methods of neuroscience are being used to get a direct understanding of how people’s economic and consumer behaviors are impacted by the way services and products are presented [refer to Sánchez-Fernández et al. (2021) for an overview over history, methods, and findings of consumer neuroscience]. It has been shown that established brain markers of decision-making impact economic choices. For example, the activation ratio between the left and right frontal hemispheres is correlated with how well advertisements are received (Ohme et al., 2010), signaling of subjective value *via* dopamine neurons is related to price sensitivity (Schelp et al., 2017), the orbitofrontal cortex is crucial for comparing subjective values in economic decisions (Padoa-Schioppa and Conen, 2017), as well as adding the factor of a social norm to the decision process (Apps and Ramnani, 2017), and the dorsolateral prefrontal cortex has been related to different cognitive biases, which have proven crucial for economic choices (Bogdanov et al., 2017; Ballard et al., 2018).

The neurophysiological method of electroencephalography (EEG) is particularly useful for investigating complex decision situations in real-life decision scenarios [refer to Ayaz and Dehais (2021) for a recent overview of recording human brain function in everyday life situations]. While its spatial resolution is inferior to neurocognitive methods like functional MRI (fMRI), it can track the neural response to environmental stimulation by measuring millisecond-scale electric fluctuations at the scalp. This is important to grasp the flexibility of real-world decision environments and identify crucial points associated with specific phases of the decision process (e.g., Van Maanen et al., 2021). Furthermore, the decomposition of the raw EEG signal into slow and fast wave components allows for distinct identification of metrics related to differential cognitive processes (refer to Cohen, 2014). EEG-based decision-making research found frontal asymmetry within the alpha band to be related to approach and avoidance behavior (Harmon-Jones, 2003; Ohme et al., 2010; Rollwaage et al., 2017), an increase of midfrontal theta, alpha, and beta as a response to gains rather than losses (Marco-Pallerés et al., 2008; Cohen, 2016), beta as signaling unexpected outcomes that can lead to an adjustment of future decisions (HajiHosseini et al., 2012; Marco-Pallerés et al., 2015; Yaple et al., 2018), and frontal theta to be related to explorative search, as well as decisions under uncertainty (Cavanagh and Frank, 2014; Demeter, 2016).

Although the field of neuroeconomics produced many promising findings, including insights into EEG markers of complex choice behavior, most real-world scenario research still puts experimental control before ecological validity. For example, even in decision-making simulations that aim to be realistic, choice options are typically limited and can be learned by participants over the course of the experiment, participants’ focus is steered toward one or a set of clear guidelines on which basis the decision should be made, and certain outcomes revealed to the participant at some point in the experiment are measured as success, failure, or some other standardized criterium (Busemeyer et al., 2006; Davis et al., 2011; Newell and Lee, 2011; Padoa-Schioppa and Conen, 2017). This degree of experimental control is justified when trying to figure out which exact aspect of the decision process certain neural response markers are related to. This is because in a messy real-world setting without set alternatives, guidelines, and decision criteria, it can be difficult if not impossible to draw clean empirical conclusions on the concrete underlying reasons behind observed effects. However, the focus on experimental control leads to decision-making environments that are hardly comparable with real-life decision-making and pose serious challenges for economists to put findings into practice (Houser and McCabe, 2014). Therefore, for a field as practically relevant as decision-making, an additional research program is necessary, which does not focus so much on a detailed mechanistic explanation of neural decision markers but allows for a more general understanding of response patterns related to certain kinds of decisions in real-world scenarios.

With the goal of a more practical approach to decision science in mind, the present work emerged from the question: “Can we, in a maximally ecologically valid setting, find markers of brain activation that are associated with certain types of decisions?” To create such an ecologically valid setting, we set out to distinguish between two simple but realistic choices: buying or not buying, without attempting to control presented stimuli or human behavior. EEG was used to measure neurophysiological responses, while participants made buying choices in a natural online shopping setting, browsing their favorite shopping application and making real buying choices in the same way they do when shopping at home. EEG was the measure of choice because of its discussed high temporal resolution, which in the present study series is particularly important as no predefined time points of interest could be determined, and its flexibility, allowing for a natural shopping experience. The metrics identified in an exploratory search over different frequency bands and common EEG markers of choice behavior are discussed in the context of previous findings on neural mechanisms of decision-making and may be explored further in both naturalistic and controlled studies on buying choices. The present research should be understood as an attempt to explore the possibility of discovering robust ecologically valid markers of decision-making with direct practical applicability, in that they can be used to distinguish between different real-world choice scenarios.

## Study 1

The goal of Study 1 was to explore EEG responses related to the decision of buying an item in an online shopping environment. The study was designed to test participants’ real-life internet buying behavior with high ecological validity. The participants were asked to use their personal mobile phones for browsing through specific categories of items in their online shopping application of choice. They were asked to simply browse naturally as they would at home and buy whatever they felt like buying on their private account. As a buying incentive, they would get a discount on all purchases. During the entire browsing and purchase process, EEG was recorded. EEG markers associated with certain aspects of decision-making have been identified across multiple EEG frequency bands and areas (e.g., Marco-Pallerés et al., 2008, 2015; Davis et al., 2011; Cohen, 2016), and the present study inherently lacked control of any specific aspect of the decision process. Therefore, the analysis was exploratory testing for differences over all 64 scalp electrodes in the theta (4–7 Hz), alpha (8–12 Hz), beta (14–20 Hz), and gamma (25–45 Hz) bands. We additionally tested differences in frontal alpha asymmetry based on findings of asymmetric brain responses, specifically in the alpha band being related to approach and avoidance behavior (Harmon-Jones, 2003; Ohme et al., 2010; Rollwaage et al., 2017). To show replicability of the exploratory results, the study was conducted in three batches during different time periods and with differing product items and participant groups. General instructions, recording, and analysis methodology, however, remained unchanged throughout all three batches.

### Study 1 Methods

#### Participants

Thirty female volunteers (mean age: 25.97 ± 4.88) participated in the first study batch for payment of ¥100 per hour and a 20% buying incentive for purchases up to ¥150. All the participants responded in a previously applied pre-screening questionnaire that they currently have the intention to buy cream and lotion products.

Sixty-two female volunteers (mean age: 26.95 ± 4.55) participated in the second study batch. The second study batch was divided into two sub-batches being recorded during two popular Chinese sales days, November 11 with 11 participants and December 12 with 51 participants. Participants from November 11 received a payment of ¥100 per hour and a 10% discount on purchases up to ¥50. Participants from December 12 received ¥75 per hour and a 20% buying incentive for purchases up to ¥400. The latter further agreed in advance to spend at least ¥180. All the participants reported a purchase history of more than 1 make-up product per month.

Forty-six female volunteers (mean age: 29.3 ± 6.09) participated in the third study batch for no fixed compensation, but a 30% buying incentive for purchases up to ¥350. All the participants reported a purchase history of more than 1 make-up product per month and an income of more than ¥8,000 per month. They agreed in advance to spend at least ¥300.

All the participants were naïve to the purpose of the study, had normal or corrected-to-normal vision, and had no history of neuropsychological diseases. After the written and verbal explanation of the task, procedure, and measurement, the participants gave their written consent.

#### Procedure and Analysis

Data collection took place at the EEG laboratory of Brain Intelligence Neuroconsulting Ltd. The participants were sat in an electrically shielded room. Their phones were used for accessing online shopping websites. Each study session lasted less than 1.5 h, with half an hour for setting up the equipment and up to 1 h of free browsing. Eye movements and a video of the participant’s field of vision were recorded using SMI eye tracking glasses (SensoMotoric Instruments GmbH, Teltow, Germany), which allow the tracking of eye movements during natural exploration of the environment. EEG was recorded with an EasyCap system (EasyCap GmbH, Herrsching-Breitbrunn, Germany). The EEG cap consisted of 64 Ag/AgCl electrodes at standard locations of the international 10/10 system, the ground electrode at position AFz, the reference electrode at position CPz, and one external electrode placed under the right eye. Impedance for each electrode was kept below 50 μΩ. The signal was digitized at a rate of 500 Hz and re-referenced offline to the average over all non-rejected measurement electrodes.

After electrode mounting, 1 min of resting-state activation was recorded, during which the participants sat still with their eyes open. Then, the participants were asked to take out their phones and open their preferred online shopping application. They were instructed to spend as long as they like browsing through the application in search of a particular type of product and buy what they liked using their own money (minus the buying incentive).

Matlab R2017B (The MathWorks, Natick, MA, United States) and the Matlab-based software package Fieldtrip (Oostenveld et al., 2011) were used for data analysis. The EEG signal was filtered with a 0.5 Hz highpass and 48 Hz to 52 Hz bandpass filter. The EEG recording was segmented into episodes according to the viewing of each product page. Artifacts were rejected *via* visual inspection. Rejected channels were interpolated using the average of their neighboring channels weighted by distance. To remove eye artifacts, principal component analysis with a logistic infomax ICA algorithm was used (Makeig et al., 2002). Given the participants were browsing the application freely, the viewing time for each product page varied naturally. The episodes were separated into two conditions: product pages of products that were purchased within the experimental session or put in the cart for possible later purchase and those of products that were not purchased. The reasoning behind making one condition out of bought products and those put into the cart was that both types reflect the general intention, eventually leading to purchase as opposed to simply discarding the item. Each episode’s EEG signal was transformed into its time-frequency representation using complex Morlet-wavelet convolution with seven wavelet cycles between 4 and 100 Hz over all channels. The frequency transform was clustered into four frequency bands of interest: theta (4–7 Hz), alpha (8–13 Hz), beta (12–20 Hz), and gamma (30–45 Hz). Furthermore, frontal alpha asymmetry was calculated as the log difference between alpha power in left-frontal (F1, F3, F5, F7, FC1, FC3, FC5, and FT7) and right-frontal (F2, F4, F6, F8, FC2, FC4, FC6, and FT8) channels (Smith et al., 2017). The peak points of each frequency band over all channels and the peak of frontal alpha asymmetry were identified for each episode. For the direct comparison of buy and no-buy trials, the time span of ±300 ms around the peak was used. Episodes with less than 300 ms around the peak points were removed from their respective comparison analysis. The remaining buy vs. no-buy episodes were averaged subject-wise. Buy vs. no-buy was compared across subjects based on the full 600 ms (i.e., 300 sampling points) by 64 channel matrix of peak time spans using a permutation-based test (Maris and Oostenveld, 2007) with 500 random permutations, with a cluster significance level of 0.05.

#### Difference Between Batches

Beyond the participants, there were only few differences between batches. In batch 1, the product type of interest was cream and lotion products; in batches 2 and 3, it was general cosmetics products. Furthermore, each batch was recorded over a short period of time, but there was a larger time difference between batches (August 2019, November/December 2019, January 2020), leading to naturally varying contents in the online shopping applications (i.e., the pool of material that can be explored) for the three different studies. Apart from this, study procedure and analysis were equivalent across all three batches of Study 1.

### Study 1 Results

#### Behavioral Results

In batch 1, the participants visited an average of 10.70 (±8.79) product pages. The mean viewing time of a page was 37.73 s (±22.21). On average, the participants bought 24.68% (±24.9) of the viewed products and put 14.21% (±16.55) into the shopping cart.

In batch 2, the participants visited an average of 14.40 (±9.99) product pages. The mean viewing time was 33.66 s (±21.73). About 21.00% (±18.95) of the viewed products were bought on average and 11.10 (±14.46) put in the shopping cart.

**FIGURE 1:**
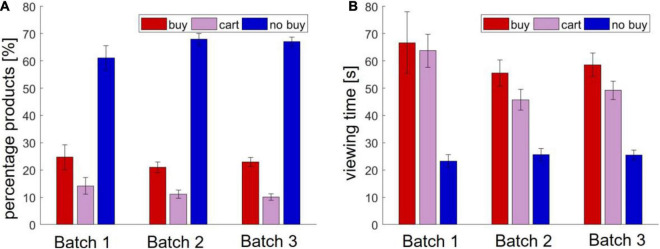
Behavioral results in Study 1. **(A)** Number of viewed and bought products **(B)** viewing time.

Finally, in batch 3, the participants viewed an average of 12.71 (±9.00) product pages for a mean viewing time of 35.80 s (±25.92). On average, the participants bought 22.91% (±19.59) of the viewed products and put 10.09% (±13.68) into the shopping cart. An overview of buying behavior and viewing time in Study 1 is shown in Figure 1.

#### Electroencephalography Results

**FIGURE 2:**
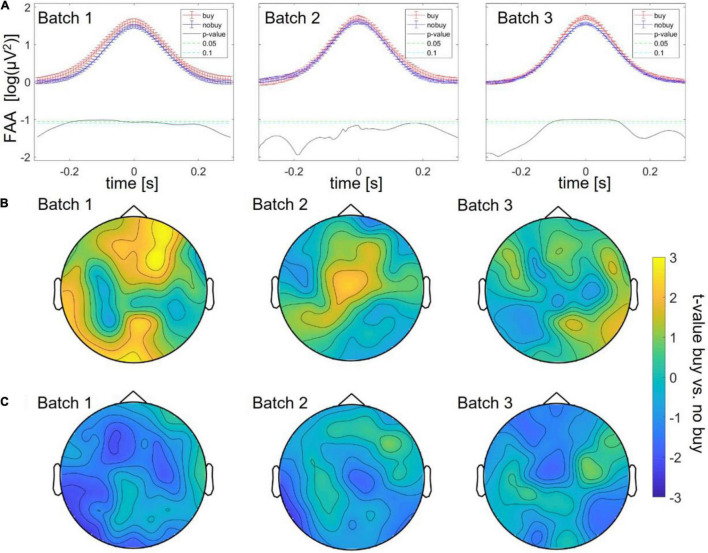
Differences between buying and no-buying trials in Study 1 divided into batches, **(A)** increased frontal alpha at frontal alpha asymmetry (FAA) peaks, **(B)** increased frontal theta at theta peaks, and **(C)** decreased occipital alpha at FAA peaks.

The EEG data were cut at the beginning and ending of each product page viewing, and the resulting trials were divided into those products that were finally bought or put into the shopping cart for later purchase (=“buying trials”) vs. those that were not bought (=“no buying trials”). Product page viewings naturally varied in duration and content of the stimulation; therefore, time points of interest through which trials of one type can be averaged and compared had to be defined. As frontal alpha asymmetry (FAA) is a typical measure of approach (Smith et al., 2017) related to choice decisions (Harmon-Jones, 2003; Ohme et al., 2010), trials were first compared around their FAA peak (right alpha power higher than left – approach) and trough (left alpha power higher than right – avoidance). In batch 1, there was a significant difference between buying and no-buying trials, with an increase in alpha asymmetry peaks for buying between 200 ms before and 100 ms after the peak (as shown in Figure 2A).

Next, an exploratory search of differences in EEG power bands at (1) respective power peaks and (2) FAA peaks was conducted. For (1) respective power peaks only theta power showed a significant increase, most pronounced across frontal channels (as shown in Figure 2B). This increase was significant with cluster correction (*p* < 0.05) for at least 20% of time points in a frontal cluster consisting of the channels AF3, AF4, AF8, FPz, F1, F2, F4, F6, Fz, FC1, FC2, and FC4 and a smaller occipital cluster consisting of the channels O1, O2, Oz, POz, Pz, P2, and P4. For (2) FAA peaks, the only frequency band that showed significant differences between the buy and no buy trials was alpha power with a decrease for buying over occipital electrodes (as shown in Figure 2C). This decrease was significant for more than 20% of the time over the channels PO7, P3, P5, P7, CP5, and CP3.

**FIGURE 3:**
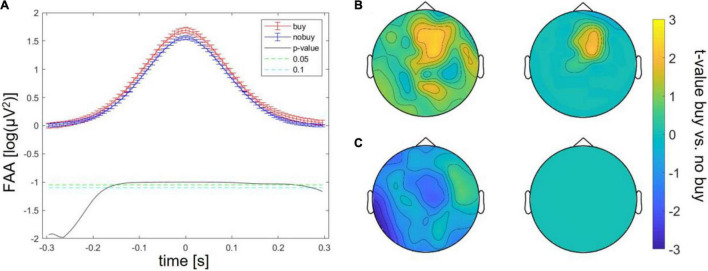
Differences between buying and no-buying trials in Study 1 with all batches combined, **(A)** increased frontal alpha at FAA peaks, **(B)** increased frontal theta at theta peaks (left: raw, right: masked by cluster corrected *p*-level < 0.05), **(C)** decreased occipital alpha at FAA peaks (left: all values, right: masked by cluster-corrected *p*-level < 0.05).

**FIGURE 4:**
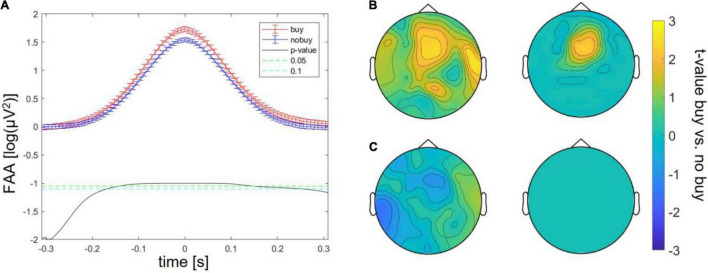
Differences between buying and no-buying trials in Study 1 over all batches with subject-wise adjustment of trial numbers **(A)** increased frontal alpha at FAA peaks, **(B)** increased frontal theta at theta peaks (left: raw, right: masked by cluster corrected *p*-level < 0.05), **(C)** decreased occipital alpha at FAA peaks (left: all values, right: masked by cluster-corrected *p*-level < 0.05).

Batch 1, therefore, revealed three significant differences between buying and no buying decisions, an increase in FAA at FAA peaks, an increase in frontal theta at theta peaks, and a decrease in centro-occipital alpha at FAA peaks. For batches 2 and 3, the same exploratory analysis was repeated. Significantly increased FAA for buying decisions could be replicated for all batches, in batch 2, between 100 and 150 ms after the FAA peak; in batch 3, between 100 ms before and 100 ms after the peak (as shown in Figure 2A). The increase in frontal theta at theta peaks could as well be seen in all three batches; however, in batches 2 and 3, it did not survive cluster correction (*p* > 0.05 for all sampling points, as shown in Figure 2B). The same was true for a decrease of occipital alpha at FAA peaks (as shown in Figure 2C). The increase in FAA for buying trials was significant over all batches taken together from 200 ms before to 200 ms after the FAA peak (as shown in Figure 3A), as was the frontal increase in theta power at theta peaks (*p* < 0.05 for more than 20% of sampling points) in a frontal cluster consisting of the channels AF7, F1, F2, F4, Fz, FC2, FC4, Cz, and Fz (as shown in Figure 3B). The difference in occipital alpha at FAA peaks was not significant with all three batches taken together (as shown in Figure 3C). No significant difference in any other frequency band was found at FAA peaks or at respective frequency power peaks in any of the batches individually as well as in all batches taken together. People bought or considered buying (put into the cart) less of the viewed product pages than they discarded (average of buy or cart decisions = 34.24 ± 2.29%). To ensure that the number of trials averaged for each subject to compare buying and no-buying EEG responses did not bias the results, we reran the analysis of all batches combined, adjusting trial numbers by randomly removing trials until the number of buy and no-buy was equal for each subject. With equal buy and no buy trial numbers for each subject, the results (as shown in Figure 4) were in line with what we found when including all trials – the increase in FAA remained significant from 150 ms before to 150 ms after the FAA peak; theta power was significantly higher (*p* < 0.05 for more than 20% of sampling points) for buying trials at the theta peak in a frontal cluster consisting of channels AF4, Fz, F1, F2, F4, FC1, FC2, FC4, Cz, C1, and C3, and the tendency of decreased alpha power for buying at the FAA peak was still present, but, when correcting for multiple comparisons *via* cluster correction, no cluster showed a significant difference between buying and no buying.

### Study 1 Discussion

The present explorative analysis revealed three markers significantly correlating with real-world buying decisions in an online shopping environment: an increase in FAA peak, an increase in frontal theta peak, and a decrease in occipital alpha at FAA peaks. FAA peak increase and frontal theta increase could be replicated across all three batches together, FAA peak also for each batch separately.

FAA peak significantly differed for buying versus no-buying choices in each of the 3 analyzed batches, both individually and taken together. FAA is an EEG metric associated with approach and avoidance behavior [as shown in Harmon-Jones et al. (2010)]. It is calculated as the log difference between right frontal and left frontal alpha power – with positive values indicating a tendency toward an approach and a positive emotional state, while negative values indicate a tendency toward avoidance and a negative emotional state. As increased alpha power is related to cognitive idling or sensory suppression of external stimulus input (Klimesch et al., 2007; Mathewson et al., 2011), this can be interpreted as increased left frontal activation being related to approach and increased right frontal activation being related to avoidance. The FAA was initially considered a trait or long-term state variable, indicating subjects’ general affective tendency and serving as a marker for affective diseases like depression. However, in recent years, it has also been frequently tested as a state variable as shown in Smith et al. (2017) for an overview. That is, some studies found that the FAA can also be related to trial-by-trial variations in stimulus input, indicating subjects’ affective stance toward the stimulus at hand (Harmon-Jones, 2003; Hu et al., 2017; Zhao et al., 2018). This has as well been shown in an economic context (Ohme et al., 2010; Ramsøy et al., 2018). However, in the present study, the timing of stimulus presentation was not controlled, as the subjects could freely search through web pages of interest. Therefore, not every point in time may be important for choice behavior. Rather, certain decision-guiding points of interest need to be identified. This may explain why not mean but peak FAA during product viewing is related to the choice of buying this same product. That is, the FAA peak reveals the maximum approach behavior a product page triggers, which may be more relevant for overall product perception than the average over the entire viewing duration (e.g., Do et al., 2008).

Frontal theta peak was repeatedly increased during buying decisions, significant over all three analyzed batches, but not for each individually. Frontal theta power has been associated with cognitive control, action monitoring, exploration of alternatives, and decisions under uncertainty (Cavanagh and Frank, 2014; Cohen, 2014; Demeter, 2016). This combination of functions fits very well with the requirements of a natural decision environment. It goes beyond a choice between a limited number of alternatives and includes the development of an – implicit or explicit – strategy on how to search within the realm of infinite options and at what point to stop the search and arrive at a decision. In an economic setting, frontal theta has been related to goal conflict in monetary choices (Neo et al., 2020). Again, this suits the idea that a practically infinite number of opportunities, increasing the risk associated with buying any one item and thereby foregoing all others, leads to frontal theta, playing a role in the binary choice of buying or not buying. Like for FAA, it is not the mean but the peak value that is of interest, as the choice is not reflected by the entire viewing duration but by decision points (Do et al., 2008).

Occipital alpha at FAA peaks is the third and only further metric that showed a significant change due to buying, however, not repeatably. Occipital alpha is related to the processing – and suppression – of external stimulus input (Rohenkohl and Nobre, 2011; Van Diepen et al., 2013). Alpha troughs represent periods of heightened attentiveness toward sensory stimulation [as shown in Klimesch et al. (2007)]. In the first batch of Study 1, we found decreased alpha power at FAA peaks, which may reflect a combination of a strong approach tendency and high sensory attention to be related to buying decisions. However, as only one of the three batches showed a significant decrease under cluster correction, the present findings do not confirm this marker’s general involvement in the buying decision process. The question under which conditions it can be found and its possible role in the purchase choice needs further clarification.

All three markers found to be related to buying choices in the present study are EEG metrics, which have been previously related to decision processes (e.g., Klimesch et al., 2007; Cohen, 2014; Smith et al., 2017). The present results, due to the lack of experimental control, may not be able to add anything to the detailed neural processes they are representing. However, they do make the important point, that these markers robustly show up in real-world buying scenarios, despite large variations in participants, products bought, and overall stimulus material. This makes them interesting candidates for practical applications, allowing us to differentiate between different choice options on a neural level. Given the robust appearance of two of the present three markers in realistic online buying choices, despite situational variations, it is now interesting to see whether they appear in any kind of buying decision, independent of situational confinements. The second study gives insights into this question by investigating the appearance of the present markers when sacrificing some of the shopping setting’s ecological validity by adding additional elements of experimental control.

## Study 2

Following three repetitions of Study 1, which demonstrated an intriguing degree of repeatability in the EEG response pattern to buying decisions despite low experimental control, Study 2 was set out to test whether EEG markers would remain the same when making a small, but a critical adjustment to the paradigm, moving it further into the direction of experimental standardization. Participants now were asked to complete 2 tasks – a free search and a controlled choice task. For the free search, they were asked to search through three predefined store pages, only looking at the product pages they were interested in and buying whatever they pleased. This first task can, therefore, be understood as another replication of Study 1, with the only difference that store pages containing the products were controlled – so the pool of possible product pages was not practically infinite. For the controlled choice, after completion of the free search, the participants were given six different products, two on each of the predefined store pages, that they were instructed to view in sufficient detail to take in all the information. All the participants viewed the same six products, except if they had already explored them during the free search; in which case, unique substitute goods were used. The viewing sequence was randomized. For each of the fixed products, the participants could decide whether they wanted to purchase the product or not. The controlled choice task, therefore, employs decisions in which the elements of choice are predefined, as is often done for the sake of experimental control. Note that, again, all purchases – both in the free search and controlled choice – were actual purchases.

### Study 2 Methods

#### Participants

About 58 volunteers (29 males, mean age: 28.49 ± 3.93) participated in Study 2 for a payment of ¥200 per hour and a buying incentive between 30 and 70% for up to ¥350, increasing stepwise with the amount of money spent. All the participants were told beforehand which kind of products they would be looking at and agreed to spend more than ¥160 buying at least three products of their own choice. All the participants were naïve to the purpose of the study, had normal or corrected-to-normal vision, and had no history of neuropsychological diseases. After the written and verbal explanation of the task, procedure, and measurement, the participants gave their written consent.

#### Procedure and Analysis

The procedure and analysis were similar to Study 1. Rather than cosmetics products, general hygiene products of no particular interest, but needed by everyone, were used. More specifically, all the subjects were shown store pages with toothpaste products, males were shown shaving products, and females were shown female sanitary products. As replication of the findings from Study 1 was intended, only peak points of metrics that have shown relevant in the comparison between buying and no-buying decisions were analyzed, i.e., frontal alpha asymmetry, theta power (4–7 Hz), and alpha power (8–13 Hz). Free search and controlled choice trials were analyzed separately to test whether replication of the fully ecologically valid results is possible in either case, i.e., with (a) predefined store pages and (b) predefined products to look at, eliminating the element of natural search.

### Study 2 Results

#### Behavioral Results

**FIGURE 5:**
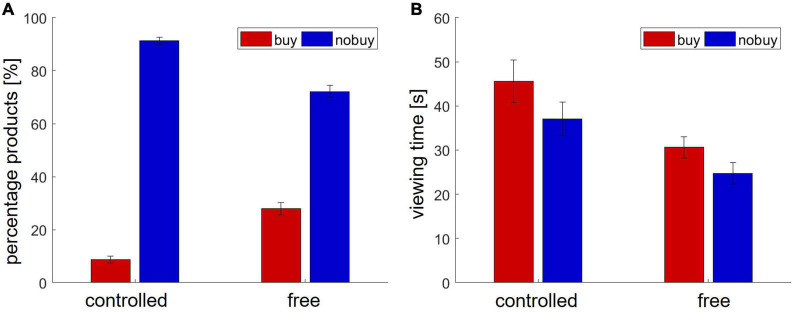
Behavioral results in Study 2. **(A)** Number of viewed and bought products **(B)** viewing time.

In the free search condition, the participants viewed an average of 13.13 (±10.72) product pages from the three predefined store pages. The mean viewing time for each page was 27.46 s (±24.44). On average, they bought 19.19% (±17.79) of the viewed products and put 8.69% (±11.72) into the shopping cart. In the controlled choice condition, each participant viewed six predefined product pages, two from each store page. They bought 8.65% (±10.33) of the viewed pages. As the buying decision had to be made right after viewing, no items were put in the cart. The average viewing duration was 27.45 s (±24.44). Figure 5 shows an overview of all behavioral results in Study 2.

#### Electroencephalography Results

**FIGURE 6:**
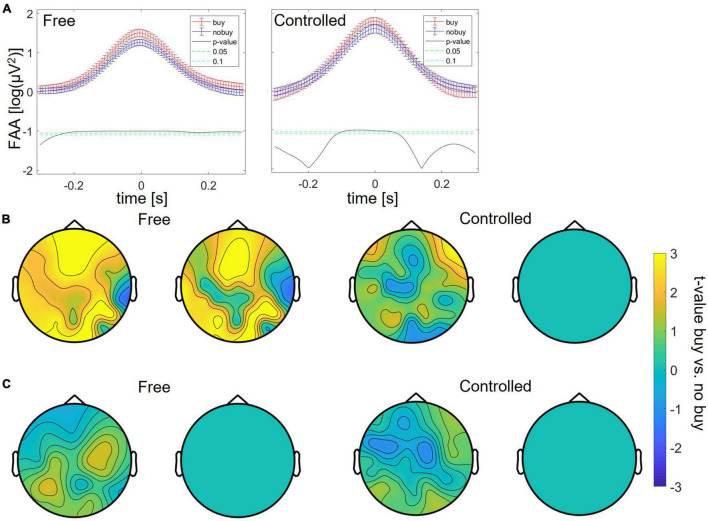
Differences between buying and no-buying trials in Study 2 for free and controlled search, **(A)** increased frontal alpha at FAA peaks, **(B)** increased frontal theta at theta peaks, and **(C)** decreased occipital alpha at FAA peaks.

Study 2 was set out to first replicate the findings of Study 1 with additional participants, different kinds of products, and uniform storefront starting points for everyone. Additionally, the controlled choice condition was added to investigate how standardization of the decision scenario can change neural responses. Here, the participants were told, rather than freely chose, which product pages to look at. In Study 2, the analysis was not exploratory, but only the three metrics identified to differ between buying and no-buying trials in Study 1 were analyzed. For the free search, the significant increase in FAA for buying trials could be replicated between 200 ms before and 300 ms after the peak (as shown in Figure 6A). Also, the significant increase of theta power at theta peaks could be replicated over a large fronto-central and occipito-central cluster (*p* < 0.05 for more than 20% of sampling points), consisting of the EEG channels AF3, AF4, Fz, F3, F2, F5, F7, FC3, Cz, C1, C2, C3, O2, PO3, PO4, PO7, TP7, T7, P7, and P8, respectively (as shown in Figure 6B). The decrease of occipital at alpha trough was not replicable (as shown in Figure 6C). For controlled search, only the increase in FAA at buying-trial FAA peaks could be replicated between 100 ms before and 100 ms after the FAA peak (as shown in Figure 6A); Figures 6B,C) show the failure to replicate even the general pattern of previously found differences in frequency power.

### Study 2 Discussion

The analysis of the second study was not exploratory but based on the three markers found in Study 1. The FAA peak increase remained significant both with a free search on predefined store pages and with controlled choices, that is, fixed products to look at and decide on. The significant theta peak increase could be replicated only for the free search condition. The occipital alpha decrease was not replicable in either condition.

Reviewing the proposed function of frontal alpha asymmetry, as an approach and avoidance signal [as shown in Harmon-Jones et al. (2010)], its robustness over each of the five batches of buying decisions is not surprising. One would assume that any decision, both in a controlled laboratory setting as in a realistic setting, would be at least partially based on approach or avoidance, or put differently, positive or negative emotions regarding the product at hand. A laboratory condition that may remove the involvement of such a metric may be the introduction of uninvolved choices; for example, if subjects are simply asked to respond to stimuli in a forced choice manner without any personal consequences to their choices (e.g., Smith, 1982; Ratcliff et al., 2018; Van Maanen et al., 2021). In this case, approach and avoidance may not differ much between decision alternatives; thus, frontal alpha asymmetry may not be an important marker. Here, choices in all five batches, including the controlled choice condition, were genuine buying decisions, with subjects spending their own money and receiving the product. Approach and avoidance may, therefore, have served as a natural guiding principle in each batch of the present study series. It is up to future studies to further clarify the role of FAA in real-world decisions by testing whether the FAA marker remains present when buying choices only reflect hypothetical purchases.

No evidence of theta increase could be found in a controlled choice setting without a self-motivated product search. As discussed, frontal theta is a metric related to explorative search strategies, uncertain choice settings, and decision scenarios involving conflicting goals (Cavanagh and Frank, 2014; Cohen, 2014; Demeter, 2016; Neo et al., 2020). This combination of functions may explain why it is only involved in buying decisions in which individuals are themselves responsible for the product search, having to explore different options, deciding when to stop searching and choosing in a self-guided manner between different alternatives.

The fact that free search requires more selective attention and suppression of sensory input than controlled stimulus presentation may explain why alpha power at alpha asymmetry peaks is only found in the free search and not in the controlled choice paradigm [as shown in Klimesch et al. (2007) for the role of occipital alpha in selective attention]. However, this metric could only be seen in one of the five batches of interest, so whether and how it is related to the natural decision process would have to be investigated by future studies.

Most importantly, the findings of Study 2 demonstrate that the neural metrics identified for realistic buying scenarios are easily disturbed due to the introduction of elements of experimental control, despite their robustness against the low-level stimulus and setting changes. Anything that makes the decision process unrealistic may change the neural processes and strategies involved in a fundamental way, leading to different output markers. In other words, even if highly controlled experimental studies can give unique insights into the neural correlates of very specific elements of the decision process, these correlates cannot easily be transferred to a real-life setting. The practically crucial question of which markers of choice behavior we can find and replicate in a naturalistic setting is, therefore, interesting in its own right and needs to be investigated in different, more ecologically valid ways.

## General Discussion

The present study series investigated neural responses to binary purchase choices in a natural online shopping setting. To assess the replicability of observed buying choice correlates despite varying sensory input and temporal patterns, the initial study was repeated three times with varying sets of participants, incentives, products of interest, and sales offers. In a follow-up study, it was examined whether the observed markers remain robust when adding a crucial element of experimental control, that is, controlling stimuli *via* predefined product pages as opposed to free search.

Three markers of real-world decision-making could be identified. Differences in FAA were robust across all decision comparisons. The increased frontal theta peak for buying was found repeatedly in Study 1 and, again, in Study 2 with free search. Without the free search element, however, no increase in frontal theta peak remained. The occipital alpha decrease was only found to be significant in one of the five batches, although tendencies remained in the other free search batches. Most importantly, the present results show that we can determine robust neural markers of decision-making, present in real-world contexts. Identifying such markers for different real-world scenarios of interest is of direct practical relevance, allowing us the distinction of different decision tendencies and strategies based on brain responses toward the stimuli in question. While the strength of controlled experimental studies lies in determining exactly which aspect of the decision process a certain marker is related to, studies of the present kind can tell us what we should actually expect to find in real-life situations.

Inferences in the present study are based on replicability – or lack of replicability – of findings over similar but, by no means, equivalent batches. Batches differ in participant age, socioeconomic status, buying incentives, and products of choice. The batches of Study 1 also differ in buying motivation, as batch 2 was recorded during popular Chinese sales days, in which product prices are strongly reduced. Many people wait to make their purchases on these days, which may result in a more focused and targeted search strategy reflected by the highest number of searched pages and shortest viewing time in this batch. Furthermore, in Study 1, all the participants were female, as the focus was on cosmetic products, but gender was balanced in Study 2, with both common and gender-specific products. All of these differences could explain variations in EEG responses between batches. However, the replicability of frontal alpha asymmetry peak increase across all batches, and both studies display overall systematic similarities regarding the decision process. Furthermore, we repeatedly find an increase of theta in batches 1–3 of Study 1 and the free search of Study 2, while it could not be replicated in the controlled search of Study 2. Batches 1–3 differ just as much from each other as they do from Study 2, but controlled and free search in Study 2 used exactly the same participants. This speaks for the fixing of the product pages to be a reason for the lack in theta increase.

Arguably, more problematic than differences between batches are uncontrolled variations in the characteristics of buying and no-buying trials. Those can and do cause comparison trials over all batches to differ in systematic ways other than the purchase decision. One difference is that the subjects generally discarded more products than they decided to buy or put into the cart, leading to unequal trial numbers. To determine that none of the found metrics is based on this bias, the analysis of Study 1 has been repeated, adjusting trial numbers to be analyzed by randomly choosing the same number of no-buying trials as there are buying trials for each subject. Both the difference in frontal alpha asymmetry (albeit for a slightly shorter time span) and the difference in frontal theta power (to the same extent as with unequal trial numbers) remained significant. Also, the non-corrected pattern of a decrease in occipital alpha did not change. Another difference between the buying and no-buying trials is the duration of time spent on product pages, which is, in each of the five batches, significantly longer for bought or put-in-cart product pages than discarded ones (*p* < 0.05). Note that the time spans compared in the EEG analysis were always equivalent – 600 ms around the respective metric of interest. Nonetheless, the difference in the base duration from which the analysis time span is selected may introduce a bias, which cannot be controlled, given we can only imply from the data which points are the decision points of interest. Certainly, there will be other systematic differences between buying and no buying trials, such as the appearance of the product pages or type of products, which are stimulus characteristics that influence the buying decision itself but may also lead to different EEG responses, which have nothing to do with the decision process itself. Therefore, comparing real-world decision data to findings from standardized experiments is certainly important, as only a sufficient degree of experimental control is able to identify exactly which aspect of the decision, or other cognitive processes, a neural marker is related to. The literature on neural markers found in the present study clearly suggests a relation to the decision process itself.

Identifying and understanding neural markers of everyday life decision making can help us understand the processes and motivations underlying human decisions from basic daily activities like food, activity, or purchase choices to bigger life decisions without the need to rely on subjective self-reports. Self-reports are not only difficult to obtain but also, at best, incomplete and, at worst, outrightly misleading, given our limited insights into what the true triggers for our actions are (e.g., Hilbert, 2012; Johnson et al., 2013) and flawed memory on temporally prolonged actions like arriving at a decision (e.g., Schacter et al., 2011). Neural responses, on the other hand, can give millisecond resolution insights into how decisions evolve, if only we can interpret them correctly. Current studies in behavioral decision sciences show that, in order to generalize laboratory findings to practical choices, it is important to identify the elements particular to real-world decision-making and investigate how they influence the overall decision process (Gonzalez et al., 2017; Prezenski et al., 2017). In order to identify such elements, we need ecologically valid studies like the present one.

The ambivalence between ecologically valid and standardized experiments on decision-making is not an all-or-nothing endeavor. To isolate particular aspects of the decision process carefully planned and standardized decision paradigms, which allow for the comparison of conditions that differ in only the very aspect of interest, are necessary. To understand, however, which kind of neural responses we can expect in real-world decision-making and how to interpret them in this respect, we need natural choice settings. Different balances and elements of freedom and control need to be explored and integrated to get a full picture of the neural side of the decision-making process. Studies such as the present one, allowing for fully naturalistic settings, are crucial to take findings from decision neuroscience out of the laboratory and make them usable for a better understanding of real-world decision scenarios.

## Data Availability Statement

The raw data supporting the conclusion of this article will be made available by the authors, without undue reservation.

## Ethics Statement

Ethical review and approval was not required for the study on human participants in accordance with the local legislation and institutional requirements. The patients/participants provided their written informed consent to participate in this study.

## Author Contributions

NH, KH, and RT designed the study. KH organized data collection. NH, KH, and BM analyzed the data. NH wrote the manuscript. All authors have reviewed and revised the submitted manuscript.

## Conflict of Interest

All authors were employed by Brain Intelligence Neuro-Technology Ltd. RT was the founder and CEO of Brain Intelligence Neuro-Technology Ltd. The authors declare that this study received funding from Brain-Intelligence Neuro-Technology Ltd. The funder had the following involvement in the study: The study was part of the funder’s research and development work. Therefore, the funder was responsible for study design, data collection, analysis, decision to publish, and preparation of the manuscript.

## Publisher’s Note

All claims expressed in this article are solely those of the authors and do not necessarily represent those of their affiliated organizations, or those of the publisher, the editors and the reviewers. Any product that may be evaluated in this article, or claim that may be made by its manufacturer, is not guaranteed or endorsed by the publisher.
